# Targeted proteomics links virulence factor expression with clinical severity in staphylococcal pneumonia

**DOI:** 10.3389/fcimb.2023.1162617

**Published:** 2023-04-03

**Authors:** Mariane Pivard, Sylvère Bastien, Iulia Macavei, Nicolas Mouton, Jean-Philippe Rasigade, Florence Couzon, Benjamin Youenou, Anne Tristan, Romain Carrière, Karen Moreau, Jérôme Lemoine, François Vandenesch

**Affiliations:** ^1^ CIRI, Centre International de Recherche en Infectiologie, Université de Lyon, Inserm, U1111, Université Claude Bernard Lyon 1, Centre National de la Recherche Scientifique (CNRS), UMR5308, École Normale Supérieure (ENS) de Lyon, Lyon, France; ^2^ Institut des Sciences Analytiques, Université de Lyon, Université Claude Bernard Lyon 1, Centre National de la Recherche Scientifique (CNRS), UMR 5280, Villeurbanne, France; ^3^ Centre National de Référence des Staphylocoques, Institut des agents infectieux, Hospices Civils de Lyon, Lyon, France

**Keywords:** *Staphylococcus aureus*, community-acquired pneumonia (CAP), panton valentine leucocidin, tandem mass spectrometer, multiple reaction monitoring (MRM)

## Abstract

**Introduction:**

The bacterial pathogen *Staphylococcus aureus* harbors numerous virulence factors that impact infection severity. Beyond virulence gene presence or absence, the expression level of virulence proteins is known to vary across *S. aureus* lineages and isolates. However, the impact of expression level on severity is poorly understood due to the lack of high-throughput quantification methods of virulence proteins.

**Methods:**

We present a targeted proteomic approach able to monitor 42 staphylococcal proteins in a single experiment. Using this approach, we compared the quantitative virulomes of 136 *S. aureus* isolates from a nationwide cohort of French patients with severe community-acquired staphylococcal pneumonia, all requiring intensive care. We used multivariable regression models adjusted for patient baseline health (Charlson comorbidity score) to identify the virulence factors whose *in vitro* expression level predicted pneumonia severity markers, namely leukopenia and hemoptysis, as well as patient survival.

**Results:**

We found that leukopenia was predicted by higher expression of HlgB, Nuc, and Tsst-1 and lower expression of BlaI and HlgC, while hemoptysis was predicted by higher expression of BlaZ and HlgB and lower expression of HlgC. Strikingly, mortality was independently predicted in a dose-dependent fashion by a single phage-encoded virulence factor, the Panton-Valentine leucocidin (PVL), both in logistic (OR 1.28; 95%CI[1.02;1.60]) and survival (HR 1.15; 95%CI[1.02;1.30]) regression models.

**Discussion:**

These findings demonstrate that the *in vitro* expression level of virulence factors can be correlated with infection severity using targeted proteomics, a method that may be adapted to other bacterial pathogens.

## Introduction

*Staphylococcus aureus* (*S. aureus*) is detected in 30% of the population, mostly as a nasal commensal, but also in the throat, on the skin, and in the gastrointestinal tract (Deinhardt-Emmer et al., 2018). In addition to this innocuous interaction with the human host, *S. aureus* has the potential to develop a wide range of diseases in humans, from mild infections of the skin and soft tissues to severe deep-seated infections such as bacteremia and pneumonia (Tong et al., 2015). The transition from the commensal status to the pathogenic one is attributed to both host and environment parameters and to the capacity of *S. aureus* to produce a wide diversity of virulence factors such as adhesins, toxins, innate immune evasion proteins, and other effector proteins. However, whilst all *S. aureus* strains encode a core set of major virulence factors, such as protein A, coagulase, alpha-hemolysin (Hla), and phenol soluble modulins (PSMs) (Peschel and Otto, 2013; Foster, 2019; Tam and Torres, 2019), the virulence gene content is not enough to predict the transition from commensalism to invasion and disease, nor can it predict disease severity. Moreover, although certain accessory genome-encoded virulence factors are specifically associated with certain diseases, such as superantigenic toxins (e.g. TSST-1, SEA,.) with toxic shock syndrome (Kulhankova et al., 2014; Berger et al., 2019), or exfoliative toxins with bullous impetigo and scalded skin syndrome (Brazel et al., 2021), the *S. aureus* strains containing these specific genes can also colonize the host without inducing any disease. Overall, this suggests that the expression level of these factors must also be considered. A recent study showed that assessing the cytotoxicity level and biofilm-forming abilities *in vitro* on THP1 cells can help predict mortality in *S. aureus* bacteremia in a lineage-dependent manner. Elevated cytolytic toxicity combined with low levels of biofilm formation was predictive of an increased risk of mortality in infections by strains of the CC22 but not the CC30 lineage (Recker et al., 2017). Another study showed that strains from pneumonia (community- or hospital-acquired) were more cytotoxic for neutrophils than nasal-colonizing strains (Deinhardt-Emmer et al., 2019). A study assessing the *in vitro* level of two regulatory RNA (RNAIII and *spr*D) identified a significant but marginal correlation between these markers and severity in the course of *S. aureus* bacteremia (Bordeau et al., 2016). Finally, high *in vitro* expression of Hla was associated with ventilator-associated pneumonia (VAP) whereas low Hla-producing strains were associated with ventilator-associated tracheobronchitis (Stulik et al., 2014). Overall, there have been very few attempts to determine whether variations in the expression level of the numerous *S. aureus* virulence factors could affect disease occurrence and/or severity in the course of an infection. High-throughput approaches to RNA and protein quantification are limited by cost-effectiveness and human resource constraints, impeding researchers from studying the correlation between the expression levels of a large panel of virulence factors and the severity and mortality data obtained from large clinical cohorts.

To overcome these issues, we developed a multiplexed targeted assay based on bottom-up proteomics, a technology of choice for implementing highly multiplexed protein assay methods (van Bentum and Selbach, 2021). Basically, the strategy consists in thorough selection of a list of reporter peptides issued from the trypsin digestion of a whole proteome, which are proteotypic of the proteins of interest while ensuring a high signal to noise ratio thanks to optimal ionization property. These peptides are detected during their chromatographic separation by Multiple Reaction Monitoring (MRM) deployed on a triple quadrupole mass spectrometer. This targeted mode of detection relies on the successive monitoring in quadrupole 3 of fragment ions generated by the dissociation of a given reporter peptide filtered in quadrupole 1 at the same time. Such a mode of signal acquisition combines high specificity and sensitivity of detection to high multiplexing property as many peptides can be concurrently monitored. Thus, an assay method targeting 42 virulence factors and resistance markers was herein deployed. After technical validation of the method, the approach was evaluated on a series of *S. aureus* isolates from 136 patients presenting a severe community-acquired pneumonia (CAP), all admitted to an intensive care unit. The results of this study show a decisive role of PVL in community-acquired pneumonia mortality and brings the proof of concept that strain-specific variations of the expression level of virulence proteins measurably impact the severity of infection.

## Methods

### Proteogenomic design of reporter peptides for detecting virulence factors

42 virulence and resistance factors were initially retained for designing the mass spectrometry-based bottom-up proteomics final assay (**Table S1**). This selection is the result of a first filtering during which some proteins (several adhesins, coagulase, some superantigens) could not be retained because of a too great allelic variation beyond the multiplexing capacities. Nevertheless, this list includes the majority of virulence and resistance determinants recognized as major such as the pore-forming toxins and PSMs, protein A, major superantigens, several innate immune evasion proteins, and the beta-lactam resistance effectors (Foster et al., 2014; Tam and Torres, 2019).

Skyline web tool (https://skyline.ms) was used to build MRM screening methods from the FASTA files of proteins of interest. All predicted proteotypic peptides obtained by in silico trypsin digestion and containing between 5 and 25 amino acids were retained as reporter peptide candidates for each protein. Although methionine or tryptophan residues have a propensity to undergo uncontrolled oxidations that may impair the sensitivity of the assay method (Carr et al., 2014), peptides containing these residues were nevertheless retained to expand the panel of peptides sampled. Conversely, peptides containing cysteine residues were systematically excluded to avoid the reduction and alkylation steps of the disulfide bonds, which could complicate the sample preparation protocol in the perspective of being able to implement this assay method as a routine test in a near future. As usually recommended, only transitions corresponding to Y type fragment ions were retained to build the testing methods, owing to their propensity to induce the most intense and less interfered signals. No more than 50 peptide candidates, i.e. 150 transitions, were included in each MRM method to ensure that a minimum of 8 data points were collected per reconstructed ion chromatogram.

The MRM methods were used to screen among all peptide reporter candidates designed in-silico those which were effective in the detection of the protein targets. This screening was performed from a detectability set of 20 *S. aureus* strains selected from the whole genome-sequenced biobank of the French National Reference Centre for Staphylococci (**Table S2**) among positive representative isolates of each of the virulence factors and resistance markers. A manual inspection of the raw data using Skyline tool enabled to establish an intermediate list of reporter peptides. A reporter peptide was considered putatively detected when the retention times of its respective transitions were perfectly aligned on the chromatogram and when it was detected in at least one of the 20 strains. Among the 42 virulence factors, at least 1 peptide reporter candidate was pinpointed for 33 proteins. In the specific case of PSMα1 and PSMα4 polypeptides, the short sequences and the location of arginine and lysine residues imposed the selection of only 1 and 2 peptides, respectively, that were therefore directly synthetized. For the remaining 7 proteins (**Table S1**, red character), no peak could be confidently assigned on the chromatogram, indicating that these virulence factors were either not expressed or expressed at a concentration below the detection limit in the strains of the detectability set. Given their potential relevance in pathogenesis, these proteins were maintained in the final assay. Hence, the synthetic forms of all peptides, were then monitored to accurately validate their retention time and optimize the ionization and fragmentation parameters. Since the absolute retention time of each peptide was accurately assessed, it enabled the building of a unique scheduled MRM assay method targeting 142 reporter peptides (**Table S1**).

### Strains of the analytical validation set

A series of strains was selected including clinical strains not carrying the gene of interest for the factors encoded in the accessory genome and isogenic strains invalidated in the genes of interest for the factors encoded in the core genome. The coherence of the proteomic profile (i.e. the combined variation in the expression of several factors dependent on global regulators) was also tested on the 2 regulatory factors Agr and SarA (**Table S3**).

### Strains and data of the clinical test set

Clinical isolates and accompanying clinical data were obtained from a previously published French cohort of severe CAP (Gillet et al., 2021). The clinical features retained for the present study were those representing severity and outcome in the cohort study: death, leukopenia (defined as total leukocyte count< 3 G/L), and hemoptysis extracted from the study by Gillet et al. (2021) (**Table S4**). The age-adapted Charlson Comorbidity Index score was retained as a control covariate to adjust prediction models to the baseline characteristics of the patients. Patients aged less than 3y (n=20), who were previously found to present very distinct clinical presentations and outcomes compared to older patients, were excluded. Patients co-infected with several *S. aureus* strains (n=3) were also excluded. Finally, 4 isolates were excluded *a posteriori* because they harbored a rare allelic variant of the Protein A which prevented its quantification by the detection method used (2 strains from deceased patients and 2 from patients who survived). Altogether, 136 isolates and patients were included in the final analyses (**Table S4**).

### Sample preparation and quality control samples

We chose to study both secreted, cell-wall associated, and cytosolic proteins in a unique standardized sample. Thus, 1 mL of each 8h-culture in Casein Hydrolysate Yeast Extract (CCY) was collected (uncentrifuged) and denatured at 90°C for 1h. 70 µL of 150-212 µm glass beads (Sigma-Aldrich) and 50 µL of a trypsin solution (1 mg/mL in 150 mM NH_4_HCO_3_; Porcine pancreas grade, Sigma-Aldrich) were added to an aliquot of 200 µL of bacterial cell suspension, and sonicated during 10 minutes at 50°C (Bioruptor ultrasonicator, Diagenode). Digestion was finally stopped by adding 5 µL of formic acid (Sigma-Aldrich) and the Eppendorf tubes were centrifuged at 10000 g during 5 minutes. 180 µL of the supernatant was finally transferred into 2 mL screw cap tubes (Labbox) endowed with a 250 µL glass insert.

For each assay series, 3 quality control (QC) samples were processed in parallel in order to assess the reproducibility of the workflow as revealed by the relative intensities of ribosomal protein-derived peptides, i.e. IYPGENVGR (50S protein L27), EMSVLELNDLVK (50S protein L7/L12), ALQSAGLEVTAIR (30S protein S11). These relative intensities were calculated as the ratio of the sum of the reconstructed peak areas of the 3 transitions of the most intense ribosomal peptide to the sum of the reconstructed peak areas of the 6 transitions of the other 2 ribosomal peptides. The QC samples were prepared by pooling CCY cultures of 7 *S.aureus* strains from the French National Reference Center for Staphylococci and aliquoting them in 1 mL Eppendorf tubes which were then treated as described above for the other samples.

### Targeted mass spectrometry parameters

The scheduled MRM experiments were deployed on a hybrid triple quadrupole/linear ion trap mass spectrometer 6500 QTRAP (AB Sciex, Toronto) equipped with an ESI Turbo VTM ion source operating at 550°C and using an ion spray voltage of 5500 volts. The settings of curtain gas and nebulizing gas 1 and gas 2 were adjusted to 50, 70, and 60 psi, respectively. The instrument and the data acquisition and processing were controlled by the Analyst 1.6.2 software. Unit mass resolution was fixed for Q1 and Q3 in MRM mode.

The tandem mass spectrometer (MS) was hyphenated to a liquid chromatography (LC) set-up comprising 2 binary pumps (1290 series, Agilent technologies). For each experiment, 80 µL of the trypsin digest was injected and submitted to on-line solid phase extraction on a PLRP-S, 12.5 mm x 2.1 mm column (Waters) connected to a Rheodyne 10-injection port valve. Peptides were desalted with 100% water containing 0.1% formic acid v/v (LC-MS grade, Fisher Scientific) for 3 minutes at a flow rate of 100 µL/min. After the valve switching, peptides were separated on a Waters BEH C18 column (100 mm x 2.1 mm; 3.5 µm particle size) using the following gradient: 98% water, solvent A (LC-MS grade, Fisher Scientific) containing 0.1% formic acid (v/v) and 2% acetonitrile, solvent B (LC-MS grade, Fisher Scientific) containing 0.1% formic acid (v/v) for 3 minutes followed by a 17-minute linear gradient to reach 35% of solvent B. The scheduling window was fixed at 1.2 minute around the expected retention time of the peptide targets, which kept the dwell time above 5 milliseconds and ensured at least 10 data points per reconstructed transition peak.

### Peptide and protein detection validation

MultiQuant TM 2.1 software (AB Sciex) was used to process the raw data leading to automated chromatographic peak detection for each transition signal as well as the calculation of their absolute area (MQ4 algorithm). Peak detection parameters were set as follows: a Gaussian smooth width of 2.0 point, a retention time half window of 35 seconds, a baseline subtraction window of 2 minutes, and a 50% noise level for baseline. A reference transition ratio (TR) was calculated for each peptide from the synthetic form (Equation 1), which validated effective detection of peptide surrogates during the analyses of the *S. aureus* strain collection. Only the peptides with a TR standard deviation below 20% relative to the mean TR were ultimately validated.


Equation 1 $$TR\;=\frac{Peak\;area\;of\;the\;most\;intense\;transition}{Sum\;of\;the\;peak\;areas\;of\;the\;three\;transitions}$$

### Protein quantification

It is assumed that the MS1 signal and the most intense transitions of the best flying peptides correlate with protein abundance (Ludwig et al., 2012) and therefore can be used as proxy for label-free quantification. To take into account any putative sensitivity deviation of the mass spectrometer instrument and variation of bacteria cell density in the suspension, the


Equation 2 $$Quantification=\frac{Sum\;of\;the\;3\;transitions\;of\;peptide\;quantifier\;of\;virulence\;protein}{Sum\;of\;the\;3\;transitions\;of\;peptide\;quantifier\;of\;protein\;30S-S11}$$

quantification of each virulence protein was derived from the calculated ratio between the sum of area of the three transitions of a peptide quantifier of the virulence protein and the sum of the three transitions of the ALQSAGLEVTAIR peptide deriving from 30S-S11 ribosomal proteins (equation 2). Peptides deriving from ribosomal proteins were used as internal relative standard as their expression level correlate with cell number amplification during the exponential growing phase (Scott et al., 2014; Bosdriesz et al., 2015; Hidalgo et al., 2022).

### Statistical analysis

We used visualization, correlation, and regression procedures to assess the relationships between the expression level of virulence factors, the genetic background of the isolates, and the clinical outcome of the infected patient. The anonymized data and software codes required to reproduce the results are available at [https://github.com/Mpvrd/Proteomic].

Relative protein quantities were log2-transformed before analysis to improve the interpretation of model coefficients, as this transformation allows to express coefficients as the change of the response variable per doubling of the protein quantity. To avoid obtaining infinitely negative values after transformation, relative protein quantities equal to zero were replaced with the smallest non-zero quantity for the same protein and divided by 2 before being log2-transformed. All analyses used R software v4.0.3 (R Core Team [2019]).

To visualize the variations of the virulome according to the genetic background of the strains, principal component analysis of the log2-transformed relative protein quantities was conducted. The linear correlation between the log2-transformed relative quantities of proteins was tested for significance using a Pearson correlation test.

The associations between clinical outcomes (death, leukopenia, hemoptysis) and the relative quantities of virulence factors were analyzed in logistic regression models. Univariate models were constructed separately for each protein, and multivariable models included all proteins at once to estimate their independent effect on the outcome. All models (including the separate univariate models) included the Charlson Comorbidity Index score as a covariate to adjust for patient health at baseline. To reduce the risk of model instability due to multicollinearity between protein quantities, strongly correlated proteins (Pearson correlation >0.5, p-value<0.05) encoded in the same operon were identified and only one protein of the operon, selected arbitrarily, was retained for statistical analysis. Coefficients of the logistic regression models were reported as odds ratios with 95% confidence intervals based on a normal approximation. The associations between relative protein quantities and the delay from hospital admission to death or hospital discharge were analyzed using Cox survival regression models, both univariate and multivariable, using the same predictors as the logistic regression models. Coefficients of the survival regression models were expressed as hazard ratios with 95% confidence intervals based on a normal approximation.

We examined whether groups of proteins, rather than individual proteins, collectively predicted clinical outcomes. To this aim, functionally-related proteins were grouped in categories listed in **Table S6**. For each of the previously described models, the point estimate of the pooled coefficients of proteins in a category was obtained as the sum of the coefficients of individual proteins. The variance of the pooled coefficients was obtained as the sum of the covariance matrix of the individual coefficients, as described elsewhere (Rasigade et al., 2020). The variances of the pooled coefficients were then used to construct 95% confidence intervals based on the same normal approximation as the individual coefficients. These pooled coefficients were interpreted as the collective effect of the proteins in the category on the clinical outcome predicted by the model.

## Results

### Method setting and analytical validation

**Figure 1:**
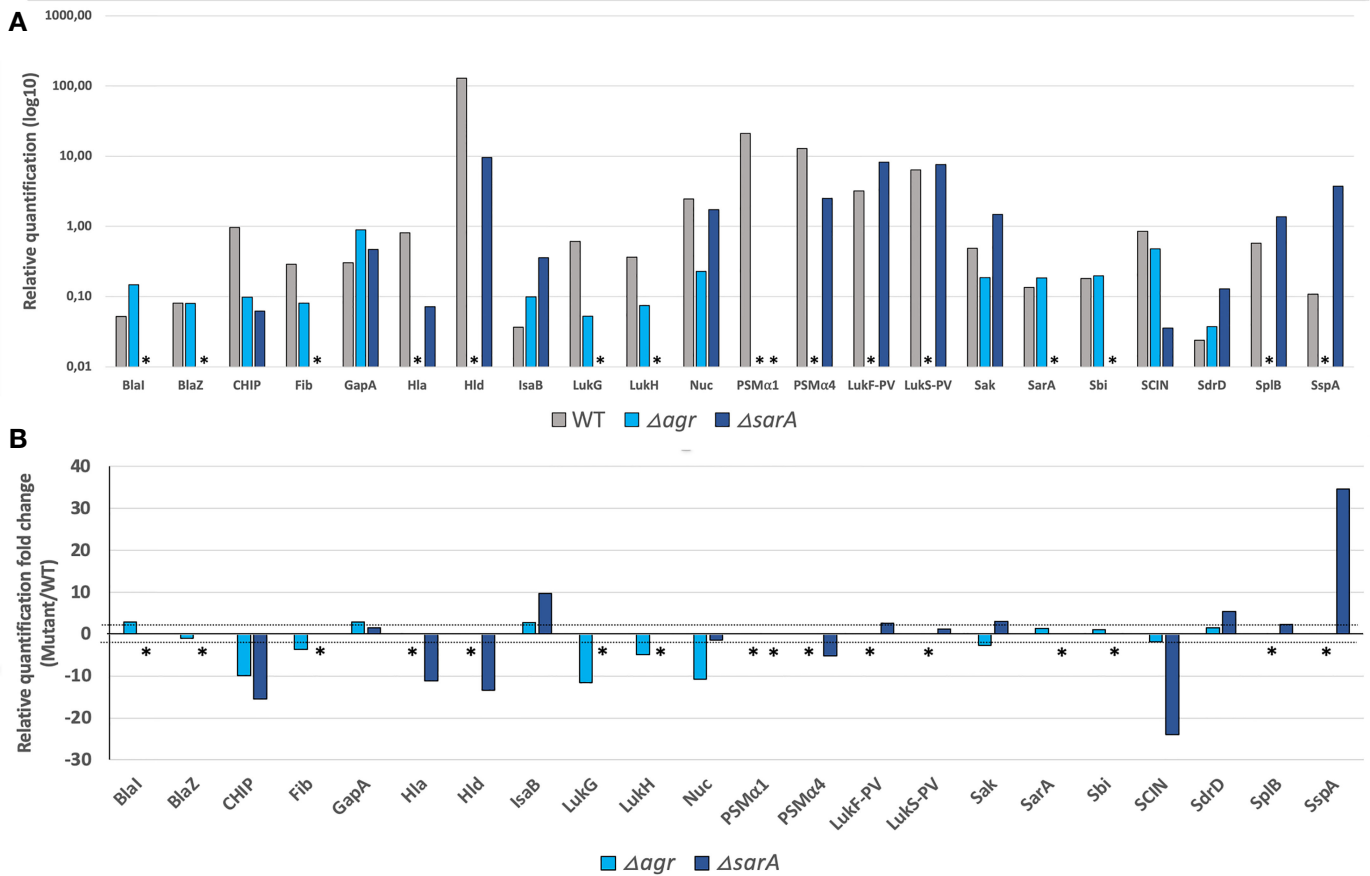
Deletions in major *S. aureus* regulators induced expected variations in proteomic profiles. Comparison of the proteomic profiles of USA300 WT (grey) and its mutants USA300 Δ*agr* (light blue) and USA300 Δ*sarA* (dark blue). **(A)** Relative amounts of the virulence factors most affected by the *agr* and/or *sar*A deletions, expressed in log10. * indicates the absence of protein detection. **(B)** Fold change of the relative quantification for the most affected virulence factors between USA300 WT and USA300 Δ*agr* or Δ*sarA*. The thresholds of 2 and -2 are represented by the dashed lines, and * indicates the absence of protein detection in mutant compared to USA300 WT.

Herein, a first step of development on a representative set of 20 *S. aureus* strains including representative isolates of each of the virulence factors and resistance markers (detectability set; **Table S2**), combined with peptide synthesis, allowed to design a mass spectrometry-based assay method targeting 142 reporter peptides used as surrogates for 42 virulence and resistance factors including the most studied candidates of *S.aureus* (Foster et al., 2014; Tam and Torres, 2019) (**Table S1**). This assay was then tested against the analytical validation set of 26 strains (**Table S3**), including clinical strains not carrying the gene of interest for the factors encoded in the accessory genome, and isogenic strains invalidated in the genes of interest for the factors encoded in the core genome, including two global regulators *agr (accessory gene regulator)* and *sar*A (**Figure 1**). This step enabled to assess the specificity of the three couples of precursor/fragments ion, i.e. transition signals, monitored for each reporter peptide in the context of true positive and true negative strain for each virulence factor (**Table S3**). Furthermore, both strains invalidated either in the *agr* or in the *sar*A locus, showed pleiotropic proteomic changes with notably for the *agr* mutant a major decrease in exotoxins and enzymes regulated by the *agr* system such as Hla, Hld, PSMα1-4, LukSF-PV, SplB (serine protease), and SspA (Glutamyl endopeptidase - V8 protease; **Table S3**; **Figure 1**) in accordance with RT-qPCR profiling (Cheung et al., 2011). In line with previous transcriptomic observations in USA300 (Zielinska et al., 2011), the *sar*A mutant strain showed a major decrease in Hld, PSMs, and Hla levels and an increase in the production of SspA (**Table S3**; **Figure 1**). It also showed a dramatic reduction in the level of BlaZ and BlaI, a phenomenon not previously identified by the transcriptomic data (Zielinska et al., 2011) and probably caused by the increased protease abundance (**Table S3**; **Figure 1**). This step also validated the choice to test unique standardized samples containing both secreted, cell-wall associated, and cytosolic proteins.

### The virulence proteome of clinical strains is highly diverse

**Figure 2:**
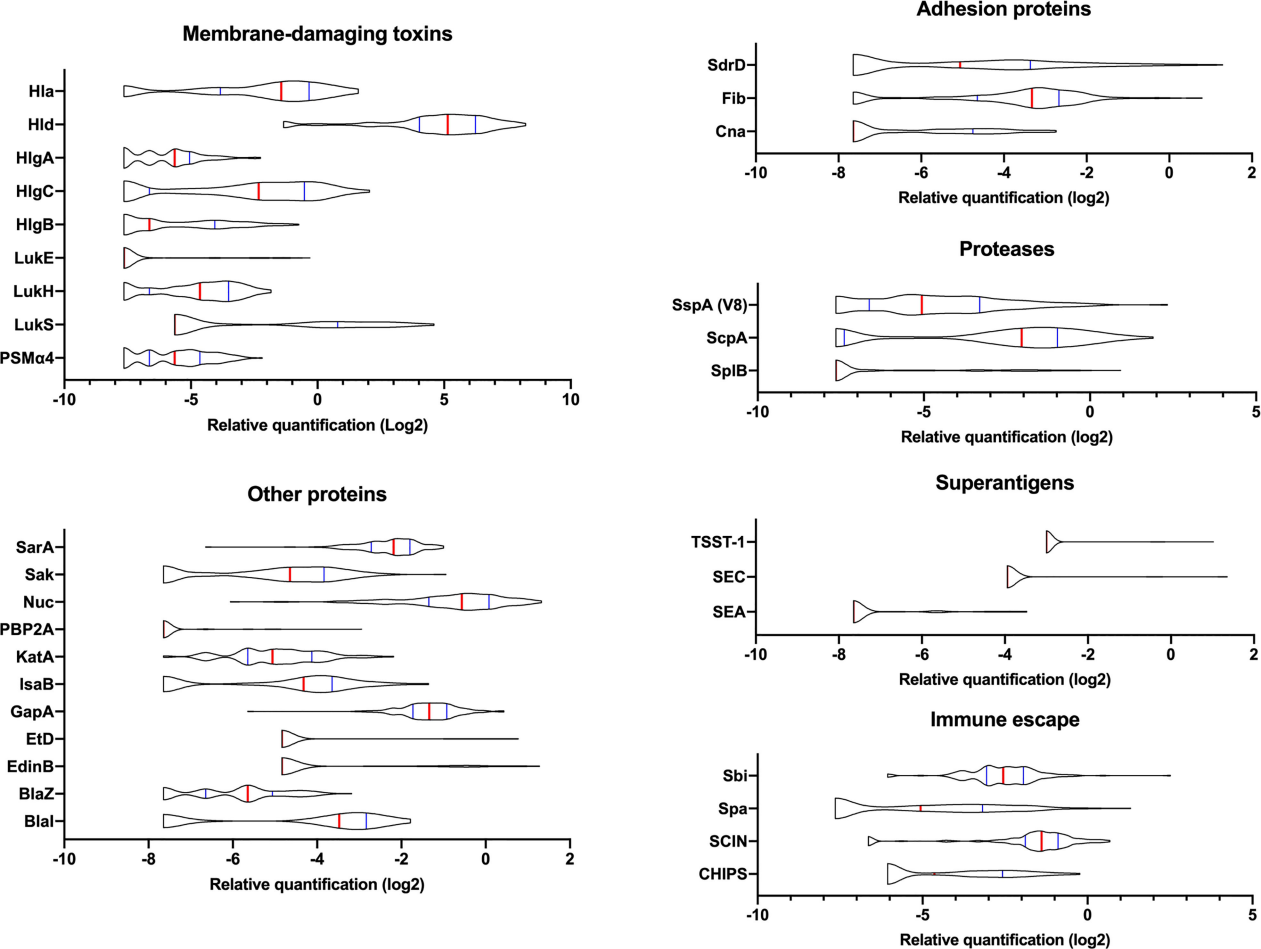
Variations in the expression of virulence factors between clinical strains. The relative amount of virulence factors expressed in log2 and categorized into functional groups are represented using violin plots. A total of 136 clinical strains were analyzed. The red vertical lines represent the medians and the blue lines represent the 1^st^ and 3^rd^ quartiles.

The 142 multiplex MRM assay was then applied to the 136 isolates from a previously published French cohort of severe CAP (**Table S4**) (Gillet et al., 2021). The 142 reporter peptides covered all potential allelic variations for each protein of the 136 strains of the test set that had been sequenced. DNA sequences are available at (Data available on ENA, PRJEB54685). The quantification of each protein was based on internal standardization relative to ribosomal peptides. Therefore, the reproducibility of the sample preparation and analytical workflow, especially the trypsin digestion step and the peak area integration, were assessed by introducing 3 quality control (QC) samples per analysis batch (**Figure S1**). The coefficient of variation (CV) calculated from the peak area ratio between the 3 ribosomal peptides monitored in all QC samples was 12%. All individual ratios remained in an uncertainty interval of less than twice the CV, hence negligible compared to the differences in the relative concentration calculated for the different virulence factors across the CAP strain cohort. Indeed, the relative quantification values of the virulence factors from the CAP strains revealed a broad variation in their expression level (**Figure 2**). However, for 5 proteins (ETA, ETB, SEB, SED, and SEH), only very few strains produced the proteins or the levels were too low to allow detection. These proteins were *a posteriori* removed from the analysis to avoid unnecessary background noise due to the lack of variance between strains, thus keeping a total of 37 factors in the analysis. As the peptide quantifier of Spa targets the repeat region of this protein, relative quantification may be biased depending on the number of repeats in the strains. However, no correlation was found between Spa expression and the number of repeats in each strain of the test set (**Figure S2**). In addition, the differences of up to 2^8^ (i.e. 256 fold, **Figure S2**) observed between strains are much higher than the 1 to 3 fold expected differences based on the number of repeats. Therefore, Spa quantification was retained in the analysis without controlling for the number of Spa repeats in the quantification values.

**Figure 3:**
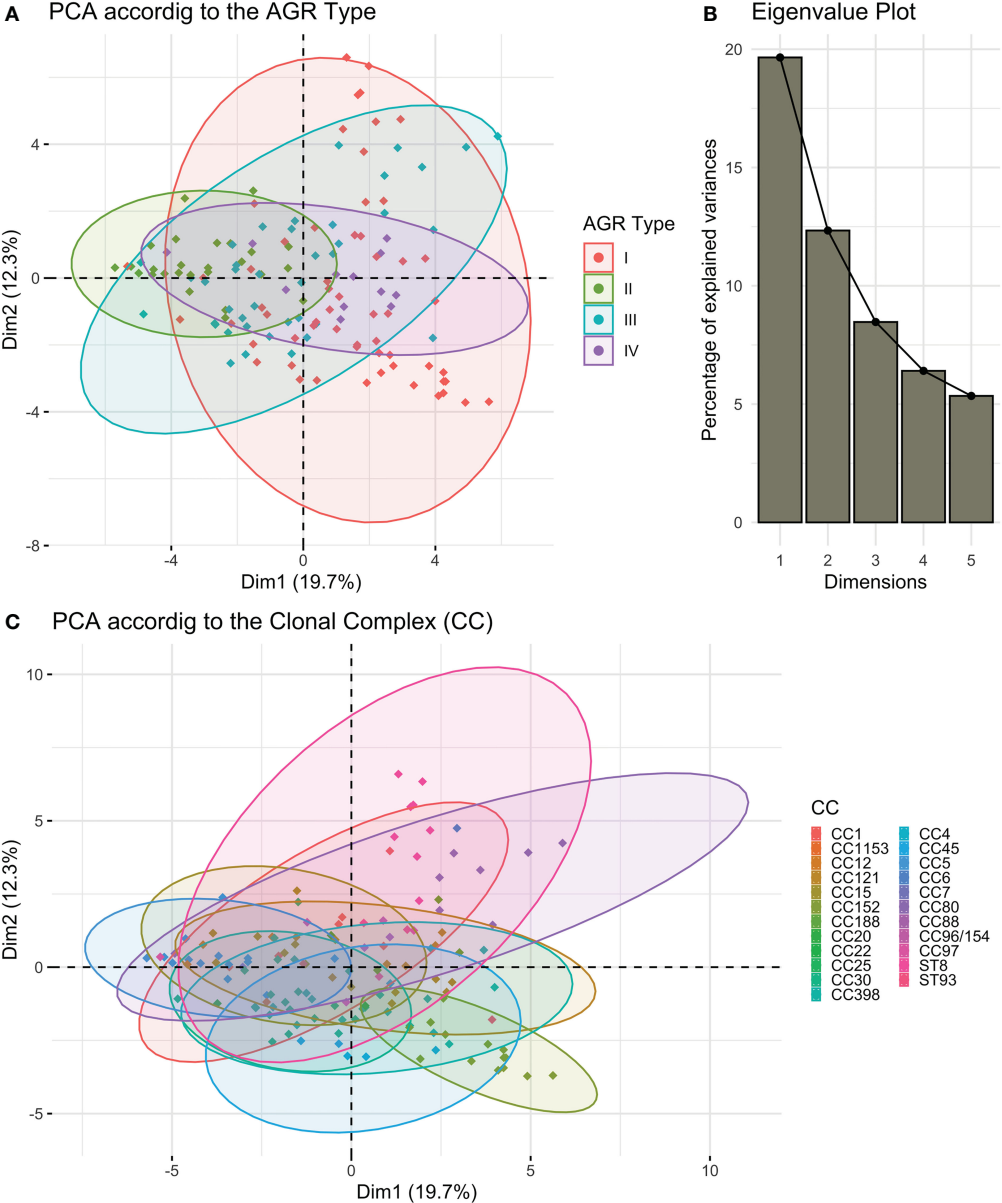
*S. aureus* proteome according to the strains’ genetic backgrounds. Principal component analysis (PCA) on the log2-transformed relative protein quantities was conducted. **(A)** The variations in the virulomes depending on the 1^st^ and 2^nd^ dimensions of the PCA analysis are visualized according to the AGR type. **(B)** The associated Eigenvalue plot for the first five dimensions of the PCA is represented using a histogram plot. **(C)** The variations in the virulomes depending on the 1^st^ and 2^nd^ dimensions of the PCA analysis are visualized according to the clonal complex (CC).

**Figure 4:**
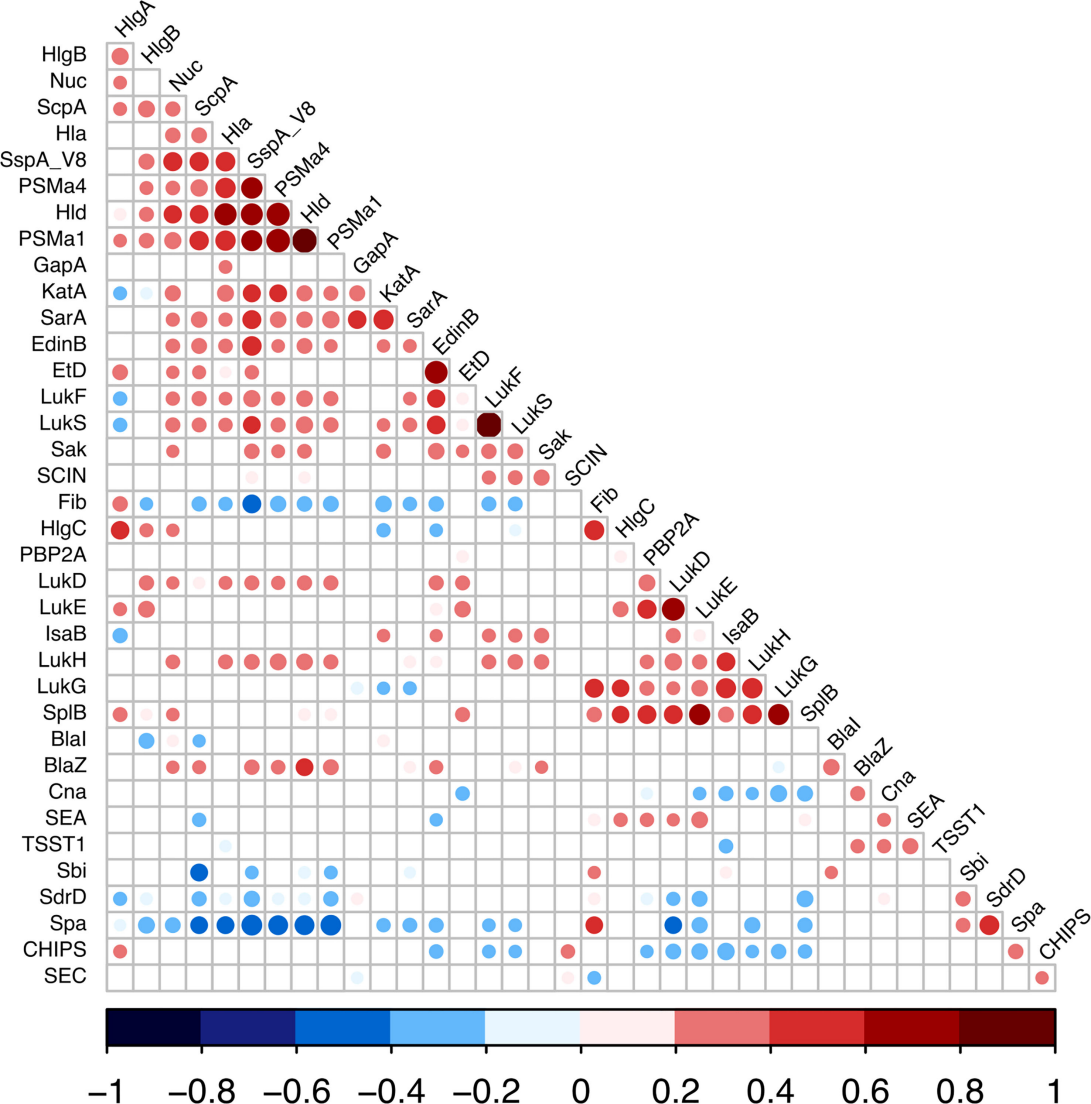
Expression correlations between *S. aureus* virulence factors. Linear correlations between the expressions of 37 virulence factors were tested using Pearson correlation test. Non-significant correlations are represented as empty squares and significant correlations as dots. The dot size is negatively proportional to the p-value. The Pearson coefficient values are represented as dark blue for negative values to dark red for positive ones.

Given the large diversity in the genetic background of the corresponding strains ((Gillet et al., 2021) and **Table S4**), the possibility that the proteome could be correlated with the genetic background was tested by principal component analysis. Neither the 4 *agr* types nor the 21 clonal complexes (CC) and 2 sequence types (ST) of the collection explained the proteome distribution, as no proteomic profile could be clearly associated with any of these genetic backgrounds (**Figures 3**, **S3**). We then searched for correlations between the 37 proteins by constructing a correlation heatmap (**Figure 4**). Strong correlations were observed for numerous virulence factors reflecting the fact that they are encoded in operons. This was the case for the LukSF-PV, LukDE, and PSMα operons. The absence of correlation between HlgC and HlgB of the HlgCB operon, however, likely reflects post-transcriptional regulatory steps on this operon (Pivard et al., 2022). Strong correlations were also observed for proteins whose expression are immediately dependent on a common regulatory pathway, as is the case for the PSMs, Hla, and Hld which are the first targets downstream of the *agr* activation pathway. Conversely some unexpected correlations were observed such as that of EdinB with Exfoliative toxin D, LukG with SplB, and Spa with SspA (V8 protease, **Figure 4**).

### PVL impacts mortality and other factors impact severity in a dose-dependent manner

**Figure 5:**
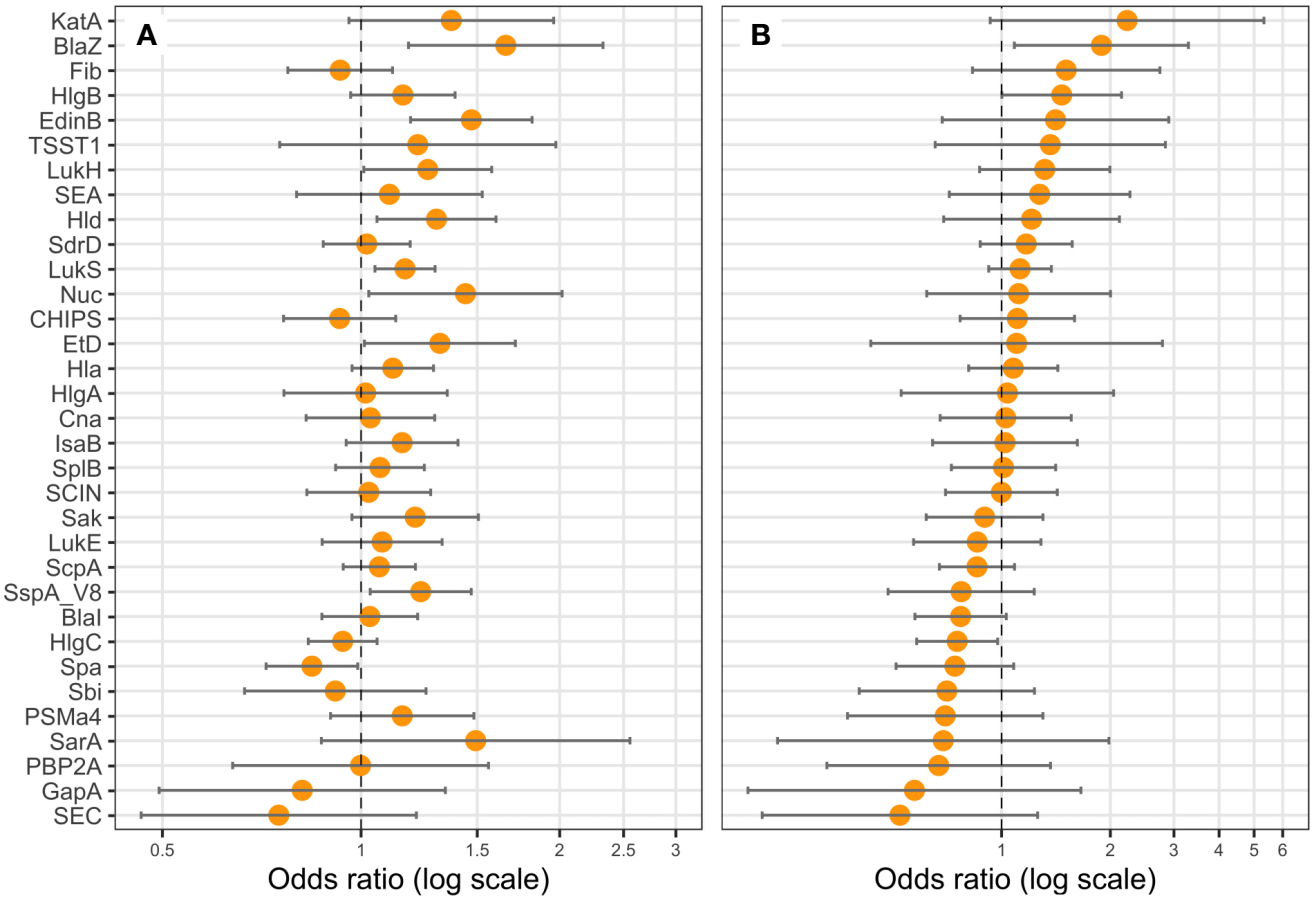
BlaZ, HlgB, and HlgC expressions are correlated with hemoptysis. Logistic regression models including the Charlson Comorbidity Index score as covariate were performed, with either the expression level of **(A)** one protein (univariate), or **(B)** all proteins (multivariate) as explicative variables, and hemoptysis as the response variable. Coefficients were reported as odds ratios (orange dots) with 95% confidence intervals (error bars). LukF-PV from LukSF-PV, LukG from LukGH, PSMα1 from PSMα1-4, and LukD from LukDE were removed from the statistical analysis to account for multi-collinearity.

**Figure 6:**
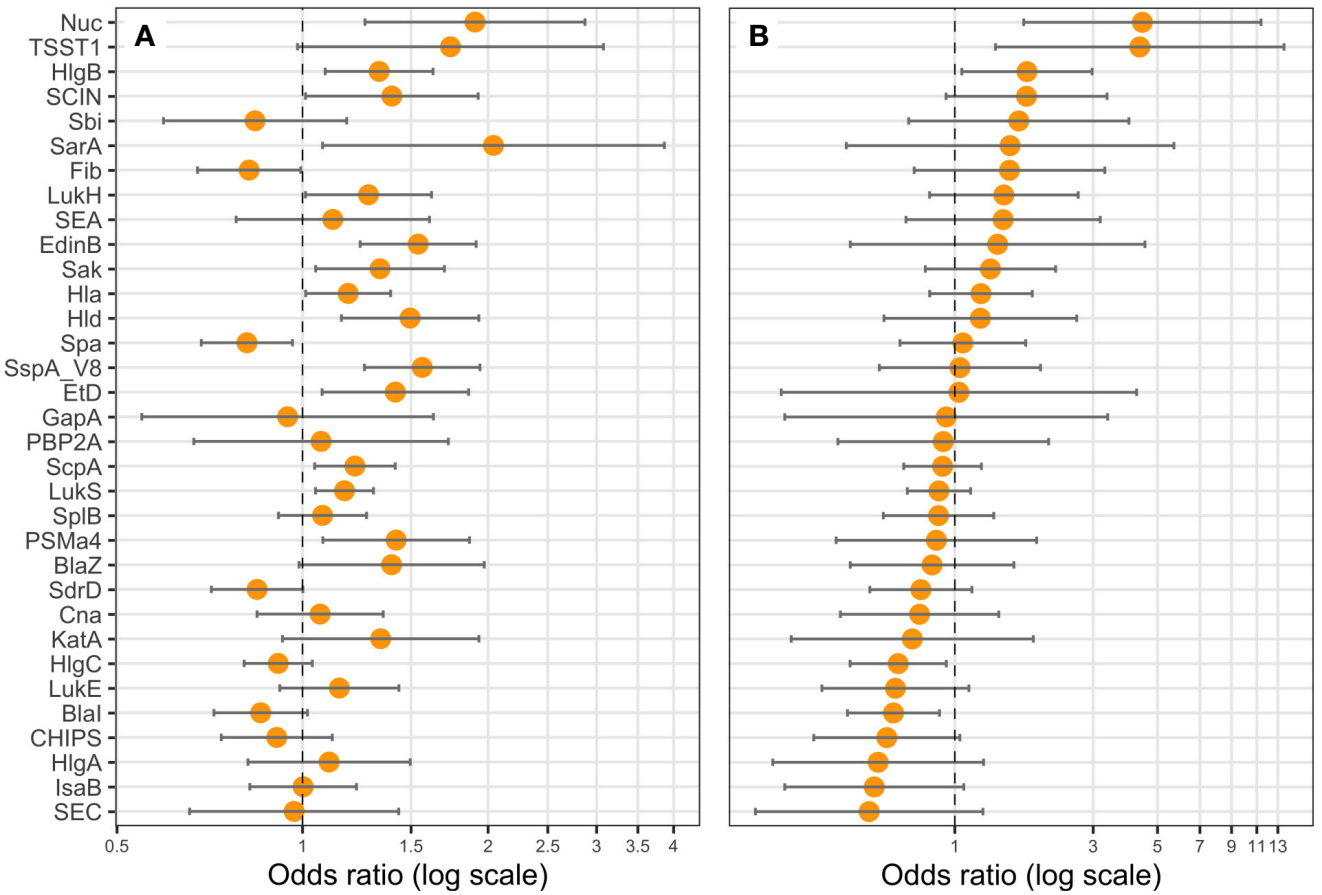
Nuc, TSST-1, HlgC, and BlaI expression are correlated with leukopenia. Logistic regression models including the Charlson Comorbidity Index score as covariate were performed, with either **(A)** one protein (univariate), or **(B)** all proteins (multivariate) as explicative variables, and leukopenia (<3G/L) as the response variable. Coefficients were reported as odds ratios (orange dots) with 95% confidence intervals (error bars). LukF-PV from LukSF-PV, LukG from LukGH, PSMα1 from PSMα1-4, and LukD from LukDE were removed from the statistical analysis to account for multi-collinearity.

**Figure 7:**
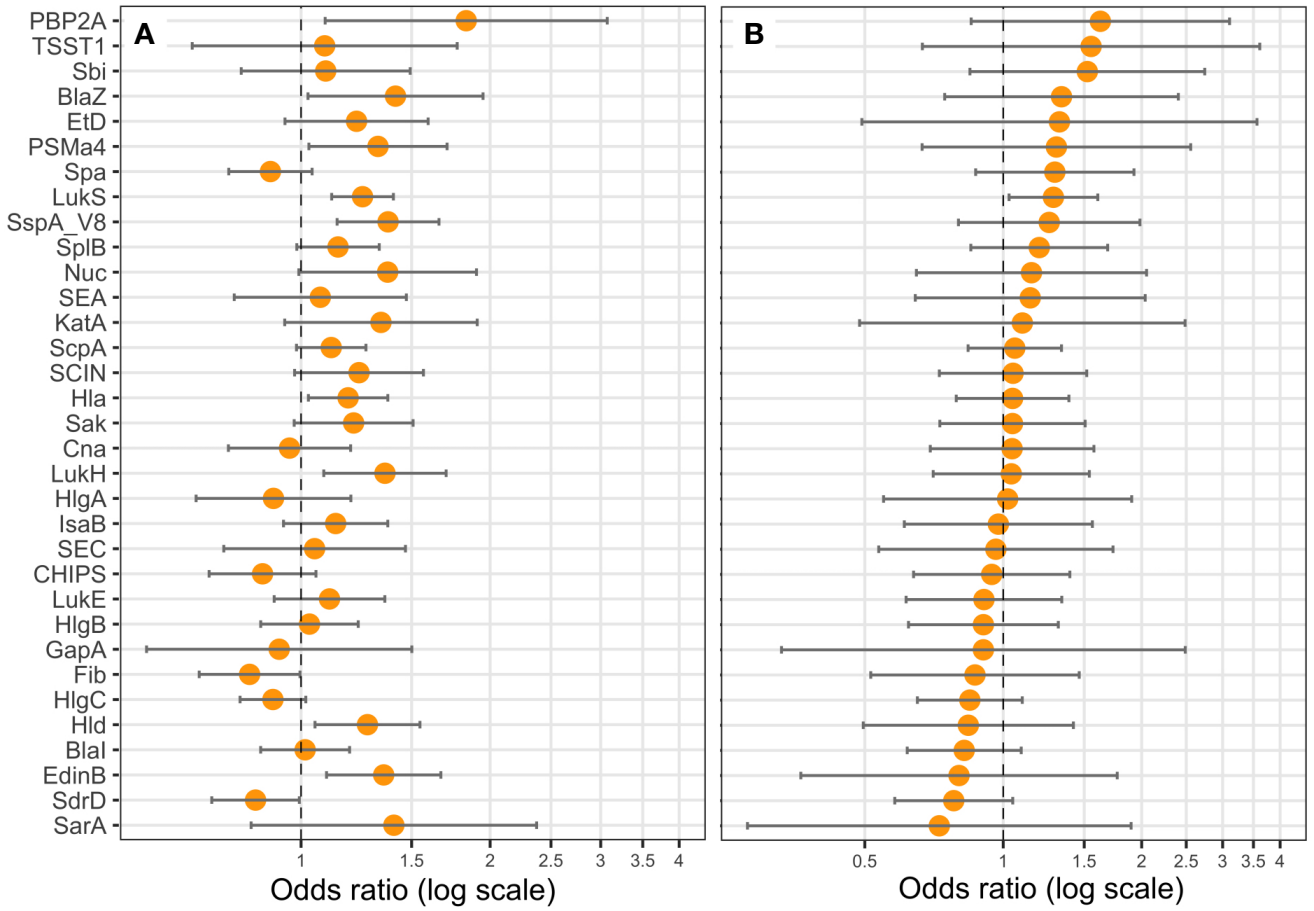
PVL (LukS-PV) expression is correlated with death. Logistic regression models including the Charlson Comorbidity Index score as covariate were performed, with either the expression level of **(A)** one protein (univariate), or **(B)** all proteins (multivariable) as explicative variables, and death as the response variable. Coefficients were reported as odds ratios (orange dots) with 95% confidence intervals (error bars). LukF-PV from LukSF-PV, LukG from LukGH, PSMα1 from PSMα1-4, and LukD from LukDE were removed from the statistical analysis to account for multi-collinearity.

**Figure 8:**
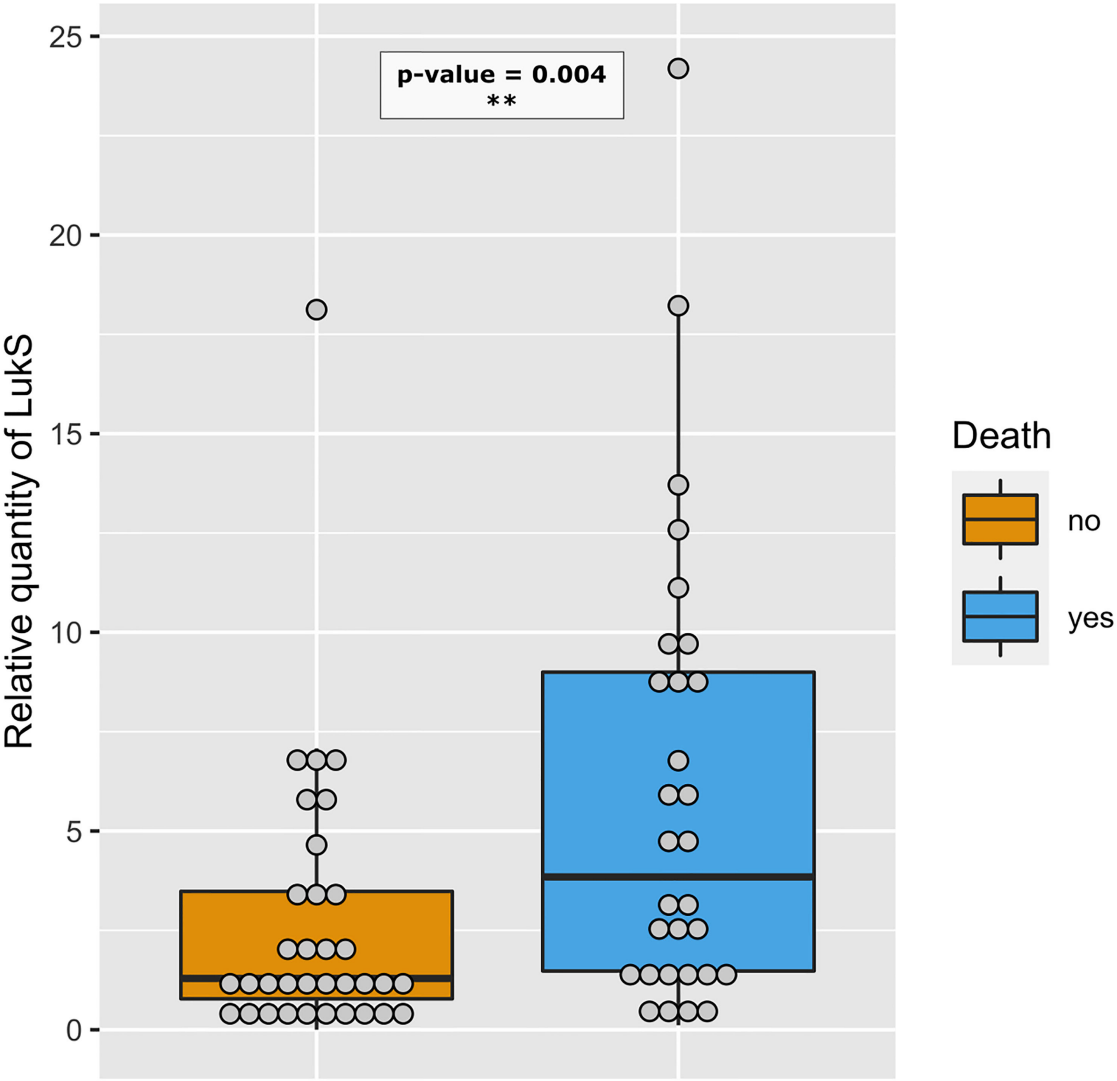
Strains isolated from deceased patients produce more PVL. Relative amount of LukS from PVL-positive strains isolated from deceased (blue, n=30) and non-deceased (orange, n=34) patients are represented by dot plots. The medians with 25^th^ and 75^th^ percentiles are represented by box plots. A logistic regression model including the Charlson Comorbidity Index score as covariate was performed, p-value< 0.01 - **.

**Figure 9:**
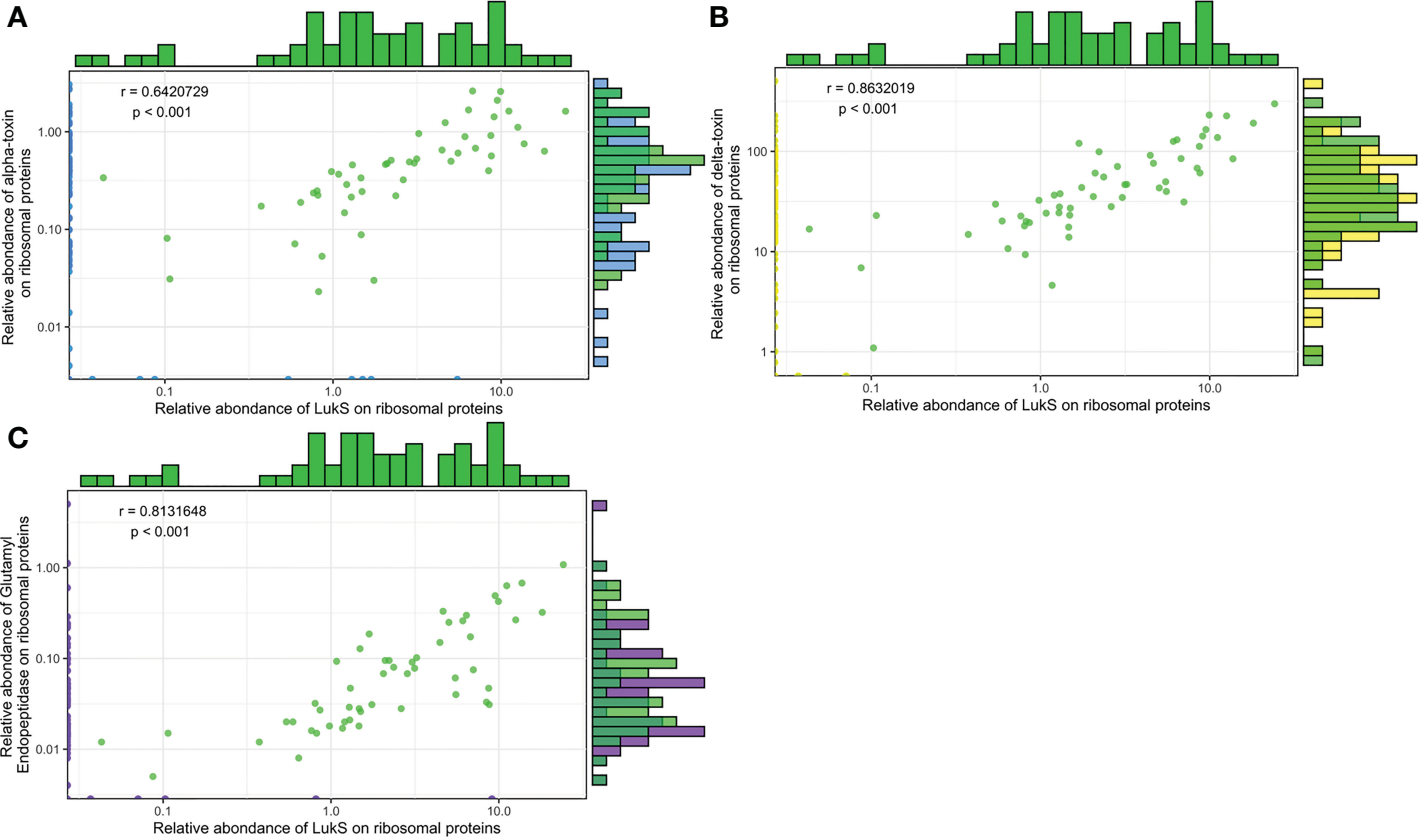
The expression levels of Hla, Hld, and SspA are correlated with PVL expression. Log2-transformed Hla **(A)**, Hld **(B)**, and SspA **(C)** relative quantities were plotted against the log2-transformed relative quantities of LukS. The density of strains expressing similar protein quantities is represented by histograms, on top for LukS and on the right side for the protein analysed (Hla, Hld, and SspA), except for null values. Green dots represent strains for which the two proteins of interest were quantified, and blue, yellow, and purple dots represent strains for which 1 of the 2 proteins of interest had a null value. A Pearson correlation test was performed.

We examined the possible association of previously identified severity parameters (death, hemoptysis, and leukopenia) (Gillet et al., 2021) with the proteomic data, assuming that such an analysis based on quantitative data could reveal novel associations, particularly regarding the virulence factors of the core genome, thus present in all strains. To increase the robustness of the analysis, we first took into account the multi-collinearity of several factors, notably those encoded in operons, except when they were not strongly correlated, as this was the case for HlgC and HlgB. Therefore, LukF-PV from LukSF-PV, LukG from LukGH, PSMα1 from PSMα1-4, and LukD from LukDE were removed from further analyses, leading to a final list of 33 proteins. This final list of factors was tested in univariate and multivariable regression, controlled on the Charlson Comorbidity Index score. The univariate analysis highlighted several virulence factors associated with severity (positively or negatively) in a dose-dependent manner. Those that were associated with hemoptysis were EdinB, PVL, BlaZ, Hld, SspA, Glutamyl endopeptidase (V8 protease), Nuc, EtD, LukH, and Spa (**Figure 5A**); those associated with leukopenia were SspA Glutamyl endopeptidase (V8 protease), EdinB, Nuc, Hld, PVL (assessed with LukS-PV), HlgB, SspP (staphopain), PSMα1-4, Etd, Sak, SarA, Hla, LukGH, SCIN, Spa, and Fib (**Figure 6A**); and those associated with death were PVL (assessed with LukS-PV), Pbp2A, SspA Glutamyl endopeptidase (V8 protease), BlaZ, Hla, PSMα (assessed with PSMα4), EdinB, LukH, Hld, SdrD, and Fib (**Figure 7A**). However, in multivariable regression analysis, only BlaZ and HlgB were positively associated with hemoptysis while HlgC was negatively associated with hemoptysis (**Figure 5B**); only HlgB, Nuc, and Tsst-1 were positively associated with leukopenia while BlaI and HlgC were negatively associated with leukopenia (**Figure 6B**); and only PVL remained associated with death in a dose-dependent manner (odds ratio 1.28 95% CI 1.02 to 1.60; **Figure 7B**). When restricting the analysis to PVL-positive strains/patients, PVL production was significantly higher in deceased versus non-deceased patients (**Figure 8**). In multivariable survival analysis, only PVL was associated with death (odds ratio 1.15 95% CI 1.016 to 1.302; **Table S5**). The expression of Hla and of several factors identified in univariate analysis was significantly correlated with the expression of PVL in PVL-positive strains (**Figure 9**), thus demonstrating that these factors were actually confounding variables. Finally, the phylogenetic tree of all strains did not reveal any clustering associated with death in patients infected with PVL-positive strains (**Figure S3**).

### Functional clustering of virulence factors

*S. aureus* virulence factors are often functionally synergistic if not redundant without necessarily targeting the same receptors. To test this hypothesis, 20/37 toxins were functionally clustered into five categories corresponding to adhesion proteins, membrane-damaging toxins, superantigenic toxins, immunoglobulin-binding proteins, and proteases (**Table S6**). These five categories were tested for their potential association with the studied severity parameters, but no significant association was found (**Table S6**).

## Discussion

In the present study, we developed a multiplexed scheduled MRM assay targeting 142 peptides used as reporters for the detection and relative quantification of a panel of resistance and virulence factors expressed by *S. aureus*. The normalization of the expression level of the virulence proteins was herein based on an internal standardization relative to ribosomal proteins, which had the advantage of dispensing with a standardization of the quantity of proteins and thus of a precise number of treated cells. Indeed, in a context of non-limiting nutrient concentration, the ribosomal protein content is fine-tuned for fitness strategy allocation, and thus exhibits a linear relationship with growth rate and hence, cell number (Scott et al., 2014; Bosdriesz et al., 2015; Hidalgo et al., 2022). The relevance and reliability of this relative quantification strategy based on the absolute signal of peptides deriving from ribosomal proteins is supported by a previous study demonstrating a good correlation between the expression level of proteins involved in various antibiotic resistance mechanisms and the resistance phenotypes of bacterial strains (Cecchini et al., 2018).

The quest for robustness, repeatability, and affordability of an assay, requires streamlining the pre-analytical sample processing as well as the analytical platform. Hence, compared to conventional protocols of bottom-up proteomics, the sample preparation was greatly simplified in the present approach. Indeed, the reduction and alkylation of the thiol group of the disulfide bond was omitted, cell lysis and trypsin digestion steps were performed concomitantly, and the time-consuming off-line or on-line peptide desalting prior to mass spectrometry analysis was avoided. Since the amount of starting biological sample is not limiting when using bacterial cultures, a robust micro-flow chromatographic-triple quadrupole mass spectrometer coupling was preferred to the gold standard nano-chromatography-high resolution mass spectrometry encountered in most proteomics experiments. Such analytical platforms are already in place in certain hospitals for various clinical purposes, some being certified to perform *in vitro* diagnostic tests.

When applied to a large series of unrelated *S. aureus* isolates, the first noteworthy feature revealed by the approach was the diversity of protein expression levels, independently of strain lineage. This is consistent with a recent study showing significant lineage-independent variability in the cytoxicity of *S. aureus* strains (Laabei et al., 2021). The coordinated expression of *S. aureus* virulence factors has been studied for decades, since the description and characterization of the major *agr* global regulon, followed by the discovery of a plethora of regulatory proteins and RNA (for review (Bronesky et al., 2016; Felden and Bouloc, 2017)). *Agr* represses the expression of numerous surface proteins such as coagulase, ClfA, FnbpAB, Spa, and others, and upregulates the expression of several toxins including Hla, PVL, certain superantigens, nucleases, and proteases (Geisinger et al., 2006; Cheung et al., 2011; Reyes et al., 2011; Xue et al., 2012). Hence, positive and negative correlations are expected within and between these two groups of factors, respectively, and were observed at the protein level in the clinical isolates of the present study. Moreover, several of the virulence factors studied herein are encoded in operons, the majority of which showed a strong positive correlation in the expression of the two proteins. This was not the case for HlgC and HlgB, suggesting that, depending on the strain, a more complex regulation may occur at the level of transcription (e.g., *hlg*B transcription generated by an alternative promoter) or translation (e.g., HlgB or C translation efficiency may vary between strains due to subtle nucleotide variations in the ribosomal binding site or upstream) (Pivard et al., 2022).

CAP due to *S. aureus* is rare but severe and is often associated with the production of a toxin rarely encountered in French isolates, PVL (Lina et al., 1999; Gillet et al., 2002). This toxin which targets the C5a receptor at the surface of granulocytes, monocytes, and macrophages (Spaan et al., 2013) was shown to impact severity of *S. aureus* pneumonia in a rabbit model (Diep et al., 2010), and is a risk factor for mortality in adolescent and adult patients with severe CAP (Gillet et al., 2021). The correlation between the observed clinical severity and the *in vitro* expression level of PVL provides additional evidence for causality and not simple mere association. However, mortality still remained important in patients infected with PVL-negative strains, who represented nearly half the cohort previously studied (Gillet et al., 2021). The contribution of other virulence factors can therefore not be dismissed, particularly since experimental studies have demonstrated the importance of Hla, Protein A, staphylococcal nuclease, and many other virulence factors in the pathogenesis of staphylococcal pneumonia (for review see (Pivard et al., 2021)). The lack of dose-dependent correlation between these virulence factors and death observed herein might reflect the dominant effect of PVL and the limited size of the PVL-negative group. Regarding the case of HlgCB, which targets the same receptor as PVL at the surface of myeloid cells (Spaan et al., 2014; Spaan et al., 2015), the former was anticipated to impact severity in a dose-dependent manner in PVL-negative strains. However, in multivariable analysis, HlgB was positively associated with hemoptysis and leukopenia whilst HlgC was negatively associated with these markers of CAP severity. These apparently conflicting results may reflect the complexity of the interplay between these toxins or may be explained by the functional duality of HlgB which composes both the HlgAB and HlgCB toxins, each having specific biological functions (Spaan et al., 2014). Lei Sun and colleagues have recently shown that HlgB alone induces the endoplasmic-reticulum-resident E3 ubiquitin ligase AMFR pathway, leading to hemoptysis in a mice model of pneumonia (Sun et al., 2023), emphasizing the potential role of the HlgB component in severity. The association of nuclease with leukopenia is remarkable since nuclease has never been shown to have a direct impact on circulating neutrophils, even though it has been shown to degrade neutrophil extracellular traps, promoting immune cell death (Thammavongsa et al., 2013). The association of TSST-1 with leukopenia observed herein is also novel in humans and could result from the extravasation of cells in a Vbeta-unrestricted manner, as demonstrated experimentally in rabbits (Waclavicek et al., 2009). The inverse association of BlaZ and BlaI with two severity parameters is in accordance with the molecular circuit of the *bla* operon since BlaI represses the transcription of *bla*Z encoding the Beta-lactamase BlaZ (Hackbarth and Chambers, 1993). Beta-lactamase hyperproduction can reduce the efficacy of penicillinase-resistant penicillins commonly used for staphylococcal infections such as oxacillin and 1^st^ generation cephalosporins (Croes et al., 2010), thus causing treatment failure (Hryniewicz and Garbacz, 2017).

The lack of association with severity when grouping 20 virulence factors into five functional categories is unexpected, as the results of animal studies have shown an additive neutralizing effect of a therapeutic antibody targeting five pore-forming toxins (Rouha et al., 2015). Altogether, these results may reflect that CAP, as opposed to VAP in which Hla plays a major role (Stulik et al., 2014), is a disease in which the main contributor to death is PVL.

Although the 42 virulence factors studied herein include the most studied candidates, this relatively low number is a main limitation of the present study. This number was dictated by technical (multiplexing capacity) and economic constraints: the need to setup a cost-effective approach that can be applied to large sets of isolates. Thus, some proteins (many adhesins, the coagulase, some superantigens) could not be included in this first version of the method because of too much allelic variation beyond the multiplexing capabilities. Moreover, we made the hypothesis that *in vitro* expression could predict an *in vivo* behavior. The fact that the expression of several virulence factors predicted death or other severity parameters in the context of CAP legitimates this approach. However, the results herein cannot conclude firmly to the absence of impact of the virulence factors lacking significant association, as the sample size relative to the number of virulence factors considered may have limited the statistical power of the analyses; moreover, the single *in vitro* condition used might not be optimal for all of them. Other culture media, possibly including various stress conditions, should be developed in future studies to improve the model. Another limitation is the choice of a cohort of patients with CAP to evaluate the potential value of this approach. The application of this method to other clinical conditions such as bacteremia, infective endocarditis, VAP, or bone and joint infections has the potential to uncover novel associations between disease presentation, complications, or outcome and the quantitative expression of virulence factors.

## Data availability statement

The datasets presented in this study can be found in online repositories. The names of the repository/repositories and accession number(s) can be found below: The proteomic data have been deposited on Peptide Atlas with accession n° PASS03811. The genomic data of the detectability and validation sets presented in the study are deposited in the ENA repository, accession number n° PRJEB60963. The genomic data of the CAP strains set presented in the study are deposited in the ENA repository, accession number n° PRJEB54685.

## Author contributions

Conceptualization: FV, JL. Methodology: JPR, MP, KM, FC, RC, JL, FV. Investigation: MP, SB, IM, NM, BY, RC, J-PR, FC, AT. Visualization: MP, SB, J-PR. Funding acquisition: JL, FV. Project administration: JL, FV. Supervision: JL, FV. Writing – original draft: MP, IL, NM, FC, FV. Writing – review & editing: SB, J-PR, KM, JL, FV. All authors contributed to the article and approved the submitted version.
