# Supplementary material for: Targeted proteomics links virulence factor expression with clinical severity in staphylococcal pneumonia
Source: Front Cell Infect Microbiol. 2023 Apr 3;13:1162617. doi: 10.3389/fcimb.2023.1162617 (PMC10106754; doi:10.3389/fcimb.2023.1162617)
Supplement: Supplementary file 1 [file DataSheet_1.pdf]

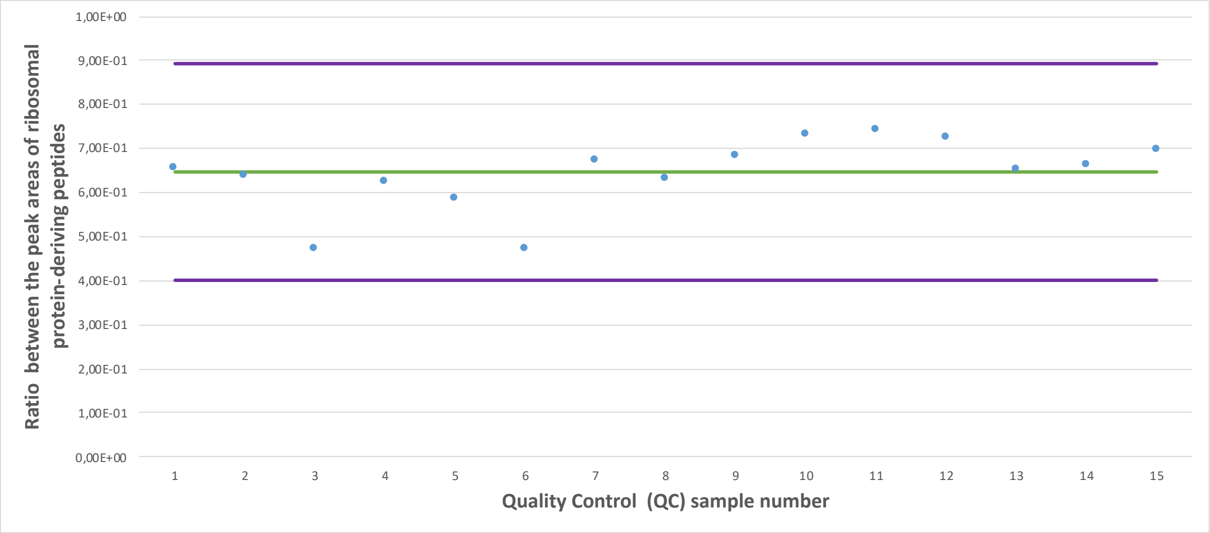

Figure S1. Monitoring of quality control samples along the analysis of *S. aureus* CAP strains. Calculated ratio between the sum of the peak areas reconstructed on the 3 transitions of the most intense ribosomal peptide and the sum of the peak areas reconstructed on the 6 transitions of the 2 other peptides, monitored in each of the QC sample. Three QCs were introduced per analysis batch of 30-33 *S. aureus* strains from severe CAP. All calculated individual ratios remained within an uncertainty range of less than twice the calculated coefficient of variation of 12%

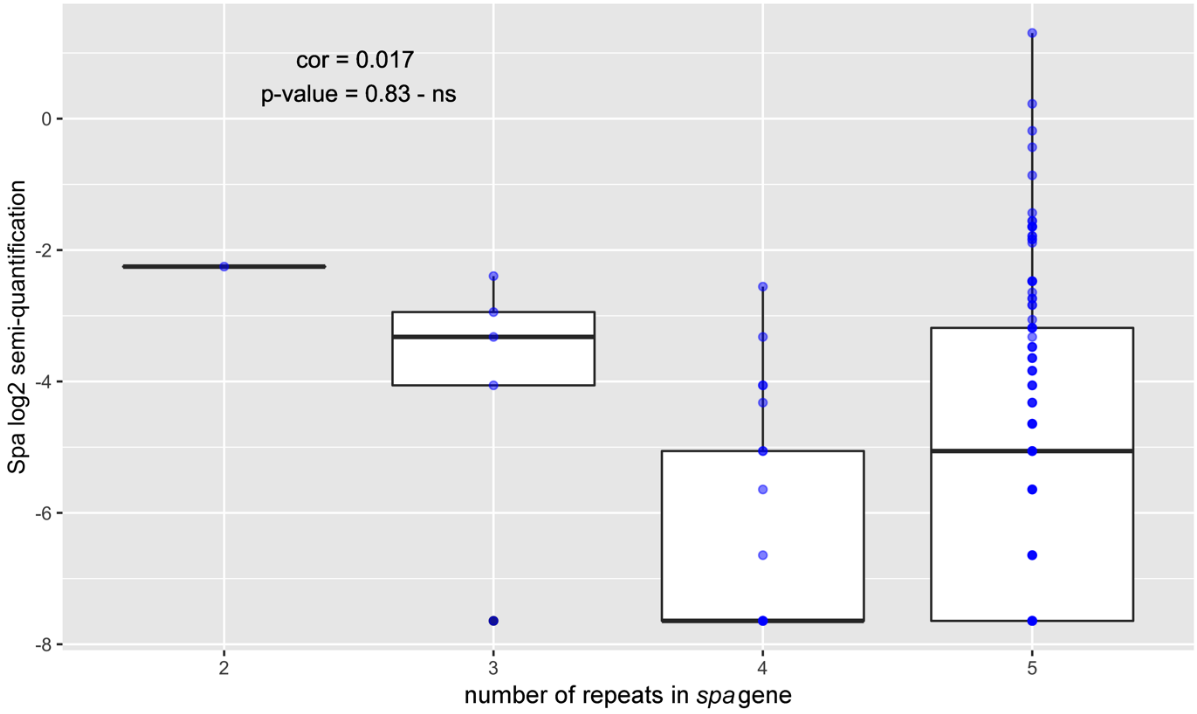

Figure S2. The number of repeats in the *spa* gene does not impact its quantification. The relative amount of log2-transformed Spa is represented by dot plots (blue dots) according to the number of immunoglobulin-binding repeats present in the *spa* gene of each strain, determined using genome sequencing. Medians with 25th and 75th percentiles are represented by box plots. A Pearson correlation test between Spa semi-quantification and the number of repeats was performed, p-value > 0.05 - ns.

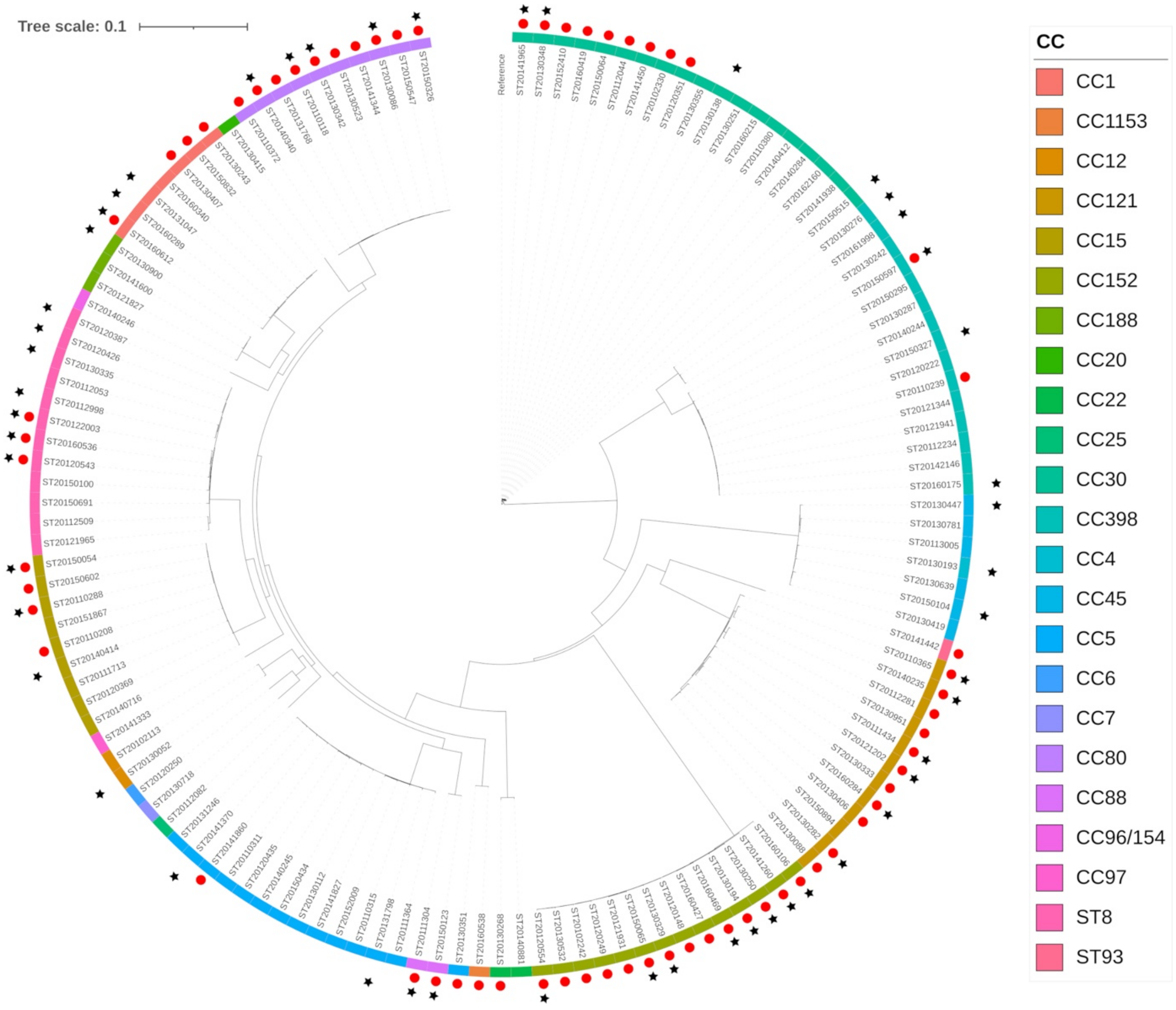

Figure S3. Rooted phylogenetic tree of all core genome SNPs of the 136 *S. aureus* strains. The tree is rooted by the *S. aureus* strain TCH60 (CC30; GenBank accession nos. NC_017342.1) using a maximum likelihood method and a generalized time-reversible model after repeat region and recombination corrections. The inner circle represents clonal complexes; red dots represent the presence of panton valentine leukocidin (PVL); the black star indicates patient death.
